# Africa’s second plan to stop mpox outbreaks through intensification, integration, and legacy

**DOI:** 10.4102/jphia.v16i4.1452

**Published:** 2025-06-26

**Authors:** Ngashi Ngongo, Abou B. Gaye, Yap Boum, Gervais Folefack Tengomo, Kyeng Mercy, Michel Muteba, Nebiyu Dereje, Rose M. Nakame, Deogratias Kakule, Raïssa Litete Beyande, Marie-Claire Fwelo, Charles Ibeneme, Senga L. Sembuche, Joshua Nyarango, Patrick C. Kabwe, Laura Ambe, Wazih N. Cho, Abdramane Diabate, Beryl Njeba, Andre Bulabula, Shanelle Hall, Yao S. Atrah, Mamadou S.K. Diallo, Fougnigué Soro, Brice W. Bicaba, Jean Kaseya, Chikwe Ihekweazu

**Affiliations:** 1 Africa Centres for Disease Control and Prevention, Addis Ababa, Ethiopia; 2 World Health Organization, Geneva, Switzerland

The World Health Organization (WHO) International Health Regulations (IHR) Emergency Committee and the Africa Centres for Disease Control and Prevention (CDC) Emergency Consultative Group (ECG) reconvened on 25 February 2025 and 26 February 2025 and both bodies unanimously recommended extending the public health emergency of international concern (PHEIC) and public health emergency of continental security (PHECS) declarations for 3 months and 6 months, respectively. This decision reflects ongoing epidemiological risks and the need for continued coordination and funding. Key factors included the increase in mpox cases, which spread to new countries in Africa (South Sudan, Sierra Leone, Tanzania) and beyond (France, Thailand)^1^ in 2025, detection of clade Ib viruses across the continent, co-circulation of clade Ia with the APOBEC3 mutation, and high population movement because of growing insecurity in Eastern Democratic Republic of the Congo (DRC). Following this, the Incident Management Support Team developed the Mpox Continental Response and Legacy Plan 2.0 (March 2025 – August 2025) that builds on the first plan (September 2024 – February 2025) and incorporates lessons learned, best practices and recommendations from the intra-action review conducted in December 2024. The plan outlines two concurrent phases: firstly, an intensification phase that focuses on immediate containment by curbing the spread and interrupting transmission chains, and secondly, a legacy phase aimed at building long-term system resilience to enhance preparedness.^2^

## Mpox response intensification

The first plan’s 6-month period initiated key interventions across 10 pillars: coordination, risk communication and community engagement (RCCE), surveillance, laboratory capacity, vaccination, infection prevention and control (IPC), case management, service continuity, research and innovation, and logistics.^3^ The new plan will build on successful practices and implement effective strategies to overcome remaining challenges. Surveillance will be enhanced by implementing digitalised community-based surveillance, managed by community health workers (CHWs) in hotspot areas where confirmed cases have been reported within the past 3 weeks.^4^ Over 3000 CHWs will be trained and deployed to support priority countries such as Burundi, the DRC, and Uganda to conduct active case findings, contact tracing and monitoring, IPC, and risk communication. To address low testing coverage, there will be a decentralisation of laboratory capacity in hotspot locations. This will involve training health professionals in primary healthcare (PHC) facilities on sample collection, transportation, and biosafety. It will also include the provision of essential point-of-care systems – such as KH Medical Radi One, SD Biosensor, and GeneXpert with cartridges – at the district level, quantitative polymerase chain reaction (qPCR) machines at the provincial or regional level, and sequencing capacity at the national level. By bringing testing closer to the cases, the requirement for sample transportation will be reduced, addressing a significant challenge faced across countries. Vaccination will be scaled up by increasing vaccine donations, securing operational funds from Gavi and partners, and adapting strategies based on vaccine effectiveness and availability. This includes combining ring vaccination around identified contacts with a targeted approach elsewhere. Case management will be strengthened through integrated training on mpox care and IPC for health professionals and CHWs, followed by supply distribution and site-level mentorship. Anticipated increases in case detection, because of enhanced community surveillance, may shift care towards home-based models, as observed in Uganda. Countries will be supported to adapt national home-based care (HBC) guidelines, train CHWs for early detection and safe isolation, and distribute essential IPC supplies to both facilities and communities. The intensification phase will include strengthening logistics and supply chain systems to support the implementation of the continental mpox emergency response plan, while ensuring continuity of essential health services.

## Mpox integration into broader health systems

The integration phase is designed to sustain mpox preparedness and reporting following the current response. Repositioning CHWs as an essential workforce for outbreak preparedness and response will necessitate the expansion of their roles and the incorporation of tools developed during the mpox outbreak response into the CHW toolkit. Mpox surveillance will be incorporated into national Integrated Disease Surveillance and Response (IDSR) strategy through Africa’s 1 million CHWs, enhancing future infectious disease monitoring. The unified mpox District Health Information Software 2 (DHIS-2) emergency management database, designed to link surveillance with lab testing, case management, and vaccination via a unique identifier, will be integrated into the national DHIS2 system for future infectious disease surveillance and outbreak response. Based on the discovery of simultaneous outbreaks of measles and chickenpox during mpox surveillance, a syndromic approach to surveillance and case management will be implemented to integrate these three diseases with similar case definitions. In addition, decentralised laboratory infrastructure, built during the emergency, will support broader syndromic diagnosis of mpox, measles and chickenpox that constitute most cases meeting the case definition of mpox. The laboratory and research teams are developing multiplex testing kits for mpox, measles, and chickenpox to enhance infectious disease surveillance integration.

## A legacy for improved readiness after mpox

Africa’s new mpox plan aims to enhance preparedness for future outbreaks by learning from past epidemics such as Ebola and COVID-19. The plan focuses on three main areas: scaling up digitalised community-based surveillance, decentralising lab testing, and increasing the local manufacturing of mpox medical countermeasures. Surveillance in Africa faces challenges, with 80% of alerts originating from health facilities, indicating reliance on passive monitoring.^8^ Delays in seeking medical help because of self-medication and traditional healers present additional difficulties. Integrating community-based surveillance with the expansion of CHWs to reach the two million CHWs target set by African leaders, could potentially create a lasting impact. Laboratory testing is a major challenge with only 44% coverage in DRC, the epicentre of the mpox outbreak.^5^ The United States (US) government’s freeze on global health funding has hindered sample transportation in DRC and Uganda. The new plan seeks to decentralise laboratory testing across all provinces and hotspot districts, eventually covering all other districts. This initiative is designed to provide nationwide access to laboratory testing and speed up the processes of outbreak investigation, confirmation, and declaration according to the 7-1-7 target: suspected outbreaks are investigated within 7 days, declared within 1 day of confirmation, and responded to within 7 days of declaration.^6^ Access to essential medical countermeasures is still limited.^7^ Efforts have focused on research related to mpox therapeutics and diagnostics, stockpiling of medical countermeasures and technology transfer to Africa for local MCM manufacturing. A few point-of-care and rapid diagnostic tests have been validated and are ready for the market, and similar efforts will continue for vaccines and therapeutics.

## A transformational plan unlike any other

The second mpox plan focuses on intensification, integration, and legacy during the response phase, aiming to transition from crisis to system resilience. It seeks to accelerate efforts to end the outbreak and prepare for future public health emergencies. The success of this plan depends on a budget of $429 million, with $196 million available from previous pledges and a funding gap of $224 million. It relies on global solidarity with shared accountability and adequate resources from all key stakeholders, including member states and partners. Only then can we effectively address mpox outbreaks and establish a lasting legacy for epidemic preparedness and health system transformation.

The authors would like to acknowledge the Incident Management Support Team (IMST) member organisations including: Africa CDC, WHO, United Nations Children’s Fund (UNICEF), Gavi, International Federation of Red Cross and Red Crescent Societies (IFRC), Joint United Nations Programme on HIV/AIDS (UNAIDS), Red Cross and Red Crescent (RCRC), Food and Agriculture Organization (FAO), World Organisation for Animal Health (WOAH), African Society for Laboratory Medicine (ASLM), Médecins Sans Frontières (MSF), Malteser International, Action Contre la Faim (ACF), World Food Programme (WFP), OXFAM, African Medicine Agency (AMA), African Vaccine Regulatory Forum (AVAREF), African Union Development Agency (AUDA)-New Partnership for Africa’s Development (NEPAD), European and Developing Countries Clinical Trials Partnership (EDCTP), Coalition for Epidemic Preparedness Innovations (CEPI), United Nations High Commissioner for Refugees (UNHCR), AFrexim Bank, Bill & Melinda Gates Foundation (BMGF), European Union (EU), MasterCard Foundation, The Pandemic Fund, World Bank.

## References

[1] Africa CDC. Africa CDC’s emergency consultative group recommends continuation of Mpox as a public health emergency of continental security [homepage on the Internet]. 2025 [cited 2025 Apr 04]. Available from: https://africacdc.org/news-item/africa-cdcs-emergency-consultative-group-recommends-continuation-of-mpox-as-a-public-health-emergency-of-continental-security/#:~:text=Since Africa CDC’s declaration of, February 2025%2C despite response efforts

[2] Ngongo N, Ndembi N, Raji T, et al. Building systems’ resilience in the mpox outbreak response in Africa. J Public Health Afr. 2025;16(1):a875. 10.4102/jphia.v16i1.875 PMC11966670 40182752

[3] Ndembi N, Ngongo N, Foláyan MO, et al. Africa’s mpox strategic preparedness and response plan: A coordinated continental effort to boost health security. Lancet Glob Health. 2025;13(2):e191–e193. 10.1016/S2214-109X(24)00464-9 39486431

[4] Pete PMN, Biguioh RM, Njuwa FKG, et al. Unlocking the potentials of community health workers for effective control of mpox outbreaks in Africa. Pan Afr Med J. 2025;50(1):3. 10.11604/pamj.supp.2025.50.1.4562940225211

[5] Samarasekera U. WHO ramps up emergency use mpox diagnostics. Lancet Microbe. 2025;6(2):101051. 10.1016/j.lanmic.2024.101051 39622260

[6] Bochner AF, Makumbi I, Aderinola O, et al. Implementation of the 7-1-7 target for detection, notification, and response to public health threats in five countries: A retrospective, observational study. Lancet Glob Health. 2023;11(6):e871–e879. 10.1016/S2214-109X(23)00133-X 37060911 PMC10156425

[7] Sharma K, Syeda S, Shah SM, et al. Overcoming barriers to medical countermeasures: Strengthening global biosecurity. Hum Vaccin Immunother. 2025;21(1):2483043. 10.1080/21645515.2025.2483043 40132079 PMC11938316

[8] Ndongo CB, Esso L, Yopa DS, et al. Roles and challenges of community health workers in epidemiological surveillance: A qualitative study in Cameroon: Rôles et Défis des Agents de Santé Communautaire dans la Surveillance Épidémiologique: Une Étude Qualitative au Cameroun. Health RES Afr. 2025;3(2):121–127.

